# Cadaveric evaluation of the feasibility of glenohumeral joint denervation

**DOI:** 10.1186/s40634-020-00322-x

**Published:** 2021-01-26

**Authors:** Amr M. Aly

**Affiliations:** 1 Department of Orthopaedic Surgery, Université Libre de Bruxelles, Brussels, Belgium; 2 Hand and Microsurgery Unit, Division of Orthopaedic Surgery, Ain Shams University Hospital, 38 Abbasiya square, Cairo, Egypt

## Abstract

**Purpose:**

To assess the feasibility of total shoulder denervation through two proposed incisions.

**Methods:**

Total shoulder denervation was performed through an extended delta-pectoral approach and a transverse dorsal approach at the spine of the scapula. The study involved six cadavers. Course and number of articular branches from the lateral pectoral, axillary and supra-scapular nerve were documented.

**Results:**

All shoulder joint articular branches were accessible through the proposed anterior and posterior approaches. The articular branch of the lateral pectoral nerve and supra scapular nerve were present in all the specimen. Axillary nerve articular branches were variable in number but when present anteriorly were proximal to the deltoid muscular branches and posteriorly proximal to the muscular branches to the teres minor.

**Conclusion:**

Total glenohumeral denervation was feasible through our proposed anterior and posterior approaches. Enhanced knowledge of articular nerve branches could provide interventional targets for joint and ligament pain, with low risk of muscle weakness.

## Introduction

Young active individuals with glenohumeral joint osteoarthritis (GHJ OA) pose a unique management challenge. The current American Academy of Orthopaedic Surgeons guideline for clinical practice on the treatment of GHJ OA for the non-arthroplasty measures, provides weak recommendations for injectable viscosupplementation as a treatment modality, whereas there is inconclusive evidence to support the use of pharmacotherapy, injectable corticosteroids, arthroscopic procedures, or open debridement [13]. Although shoulder arthroplasty is considered the definitive treatment of GHJ OA, it is not an ideal procedure for the young patient. Young patients have a significantly higher incidence of component failure and inferior patient-reported outcomes than older ones [6].

Joint denervation has become increasingly popular as a salvage procedure for joints with chronic intractable pain in wrist and elbow joints [2, 11]. Denervation represents an appealing alternative for pain relief while maintaining joint motion, with a minimal recovery period. The denervation of a joint is an operative procedure aimed at eliminating pain through interruption of sensory nerve function [16]. The main question regarding the safety of denervation procedures is the possible side effects resulting from definitive suppression of the neural messages from the joint as well as the nociceptive inputs. Intra-articular sensation is mediated by nerve endings in the synovial membrane, where they form a plexus of delicate nerve fibrils with end-bulbs beneath the free surface. In addition to this sensory articular mechanism, there is an auxiliary sensory defence, designated as deep sensation in the adjacent ligaments and muscles, its chief function is recognition of movement and sense of position. Inability to appreciate protective impulses exposes the joints to continued and uncontrolled injury. On the other hand, when the articular sensation is involved alone, as in the division of an articular branch of the peripheral nerve, there remains a line of protection in the deep system [5]. Zamprogno et al. evaluated the effects of denervation on animal upper limb function and concluded that no alteration was detected [17].

The aim of our study was to assess the feasibility of total glenohumeral joint denervation through two proposed incisions.

## Material and methods

Our study was performed on six fresh frozen adult human cadaveric shoulders that are legally donated to our institute from our body donation program. Specimens were stored at − 20 °C and thawed at room temperature for 24 h prior to testing.

We assessed the feasibility of denervating the articular branches of the lateral pectoral and the axillary nerve through delto-pectoral approach, and the axillary and the supra-scapular nerves through a transverse approach over the scapular spine.

**Fig. 1:**
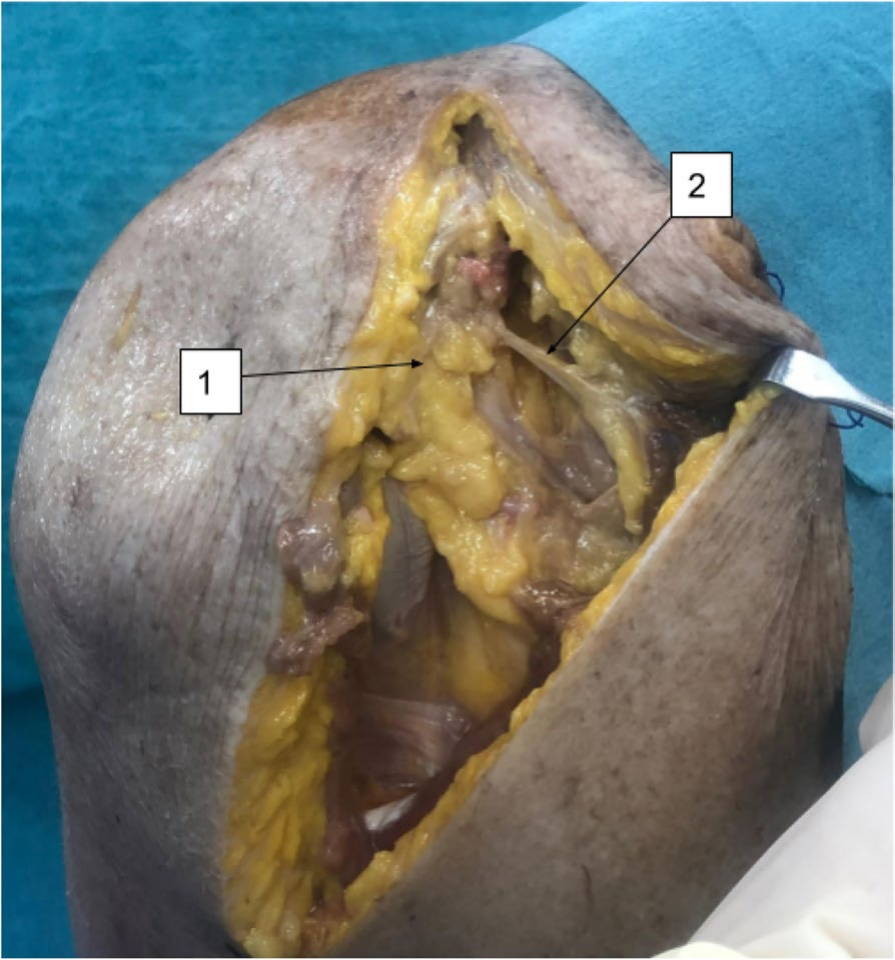
Delto-pectroal approach. (1) Coracoid process; (2) Articular branch of the lateral pectoral nerve

**Fig. 2:**
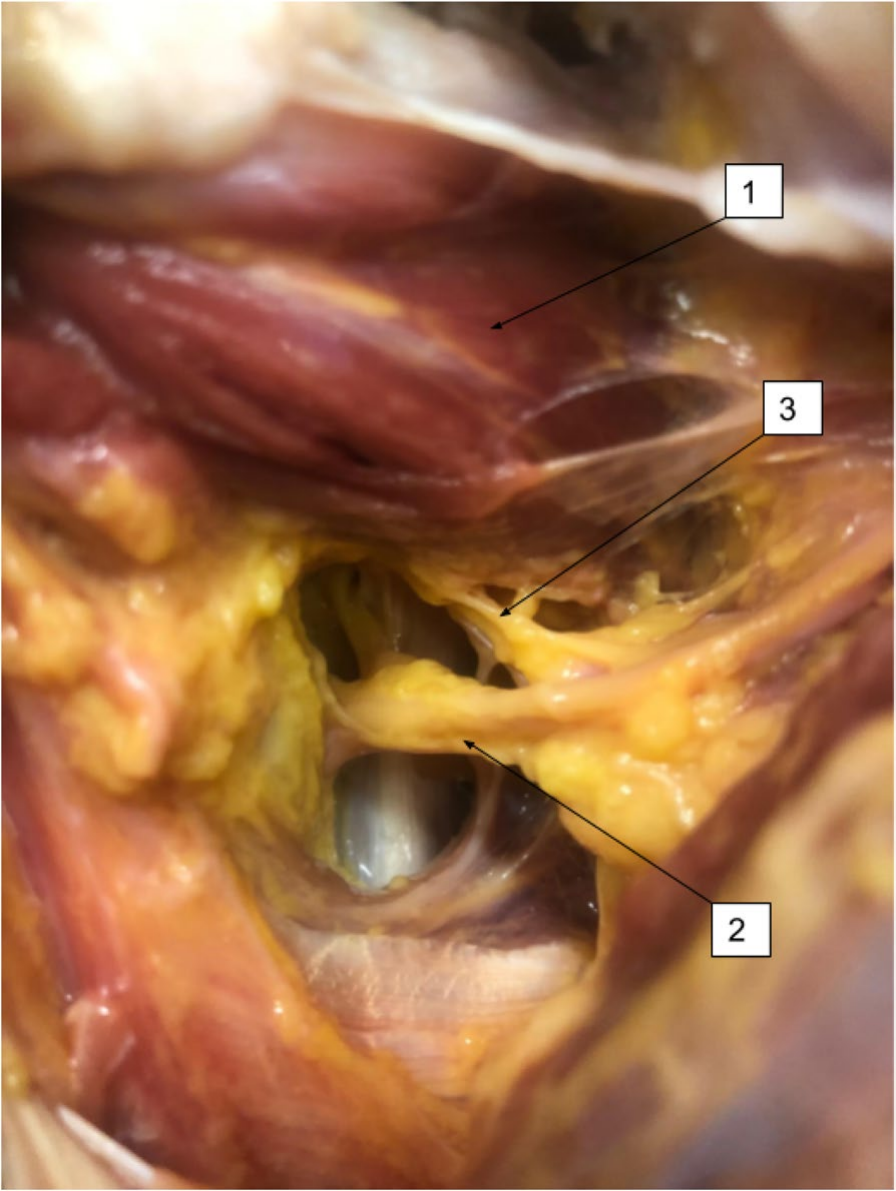
Delto-pectroal approach. (1) Subscapularis muscle; (2) Axillary nerve; (3) Articular branch of the axillary nerve

After thawing, a delto-pectoral approach was incised. Starting from the clavicle proximally till the deltoid muscle insertion distally. Demarcation of the dissection plane was facilitated by cephalic vein identification. The whole delto-pectoral interval was defined. Lateral retraction of the deltoid muscle revealed the coracoid process. Dissection medial to the coracoid process revealed the articular branch of the lateral pectoral nerve directed to pierce the corao-acromial ligament (Fig. 1). The conjoint tendon was then retracted medially. The clavipectoral fascia was incised revealing the subscapularis muscle. External rotation while placing the arm in adduction placed the subscapularis fibers under tension. The axillary nerve was identified inferior to the subscapularis tendon entering the quadrangular space. Small articular branches from the axillary nerve were identified anteriorly just proximal to the quadrangular space (Fig. 2). Identification of the superior and inferior borders of the subscapularis tendon was followed. A horizontal split is made in the tendon at the junction of the upper two-thirds and lower third. This split was moved medially till the muscle fibers. The split was then extended with curved Mayo scissors, leaving the capsule intact. With the capsule exposed by the open limbs of the scissors, a small swab was inserted medially deep to the muscle to release any present articular branch from the sub-scapular nerve.

**Fig. 3:**
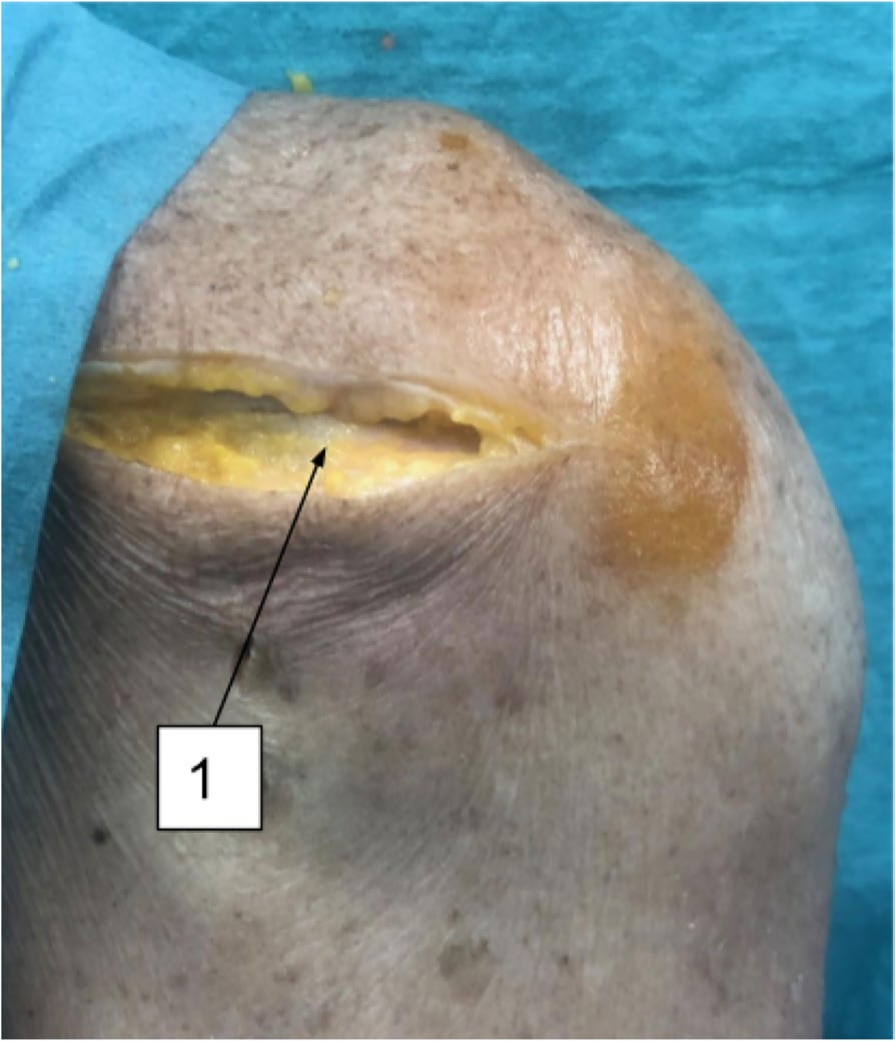
Posterior approach. (1) Spine of the scapula

**Fig. 4:**
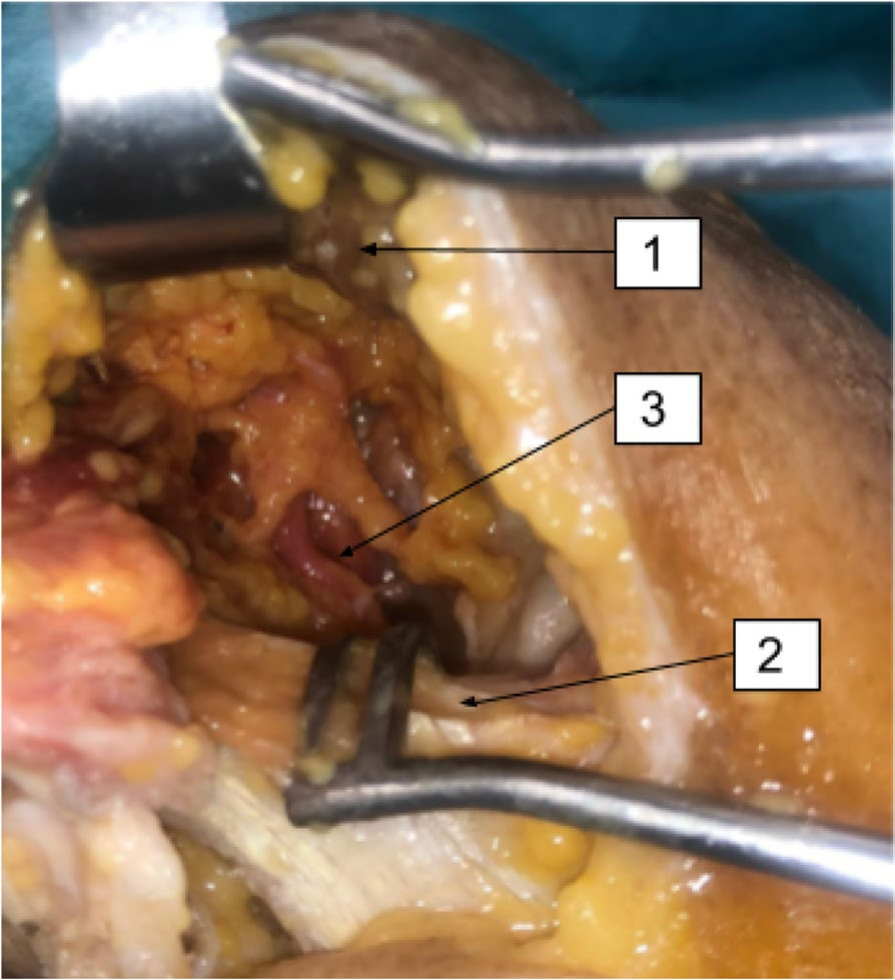
Posterior approach. (1) Supra-spinatus muscle; (2) Spine of the scapula; (3) Surpa-scapular nerve at the supra-spinatus fossa

**Fig. 5:**
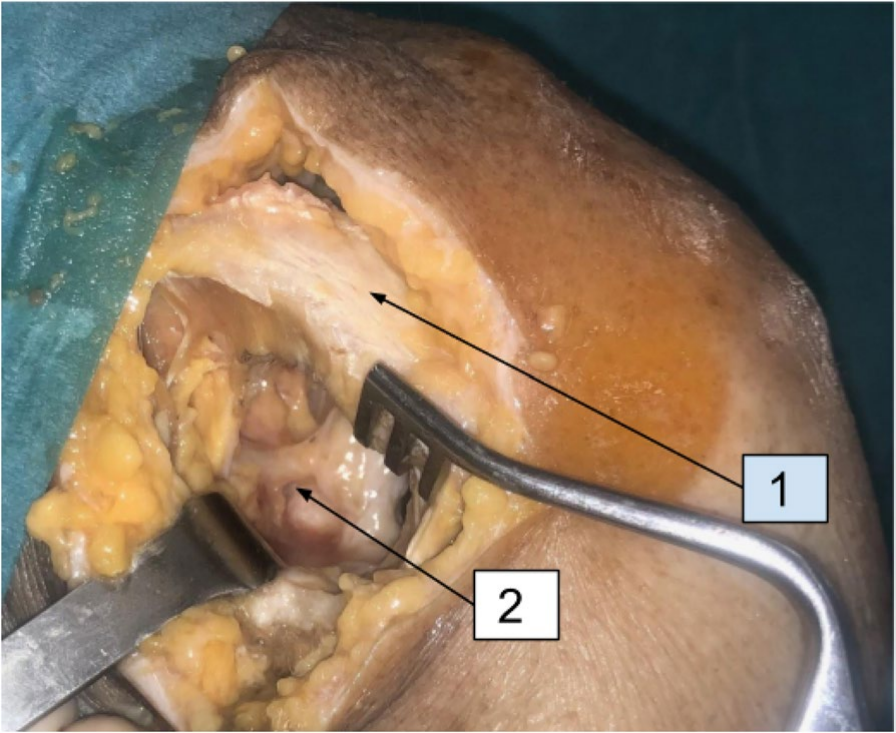
Posterior approach. (1) Spine of the scapula; (2) Surpa-scapular nerve at the infra-spinatus fossa

**Fig. 6:**
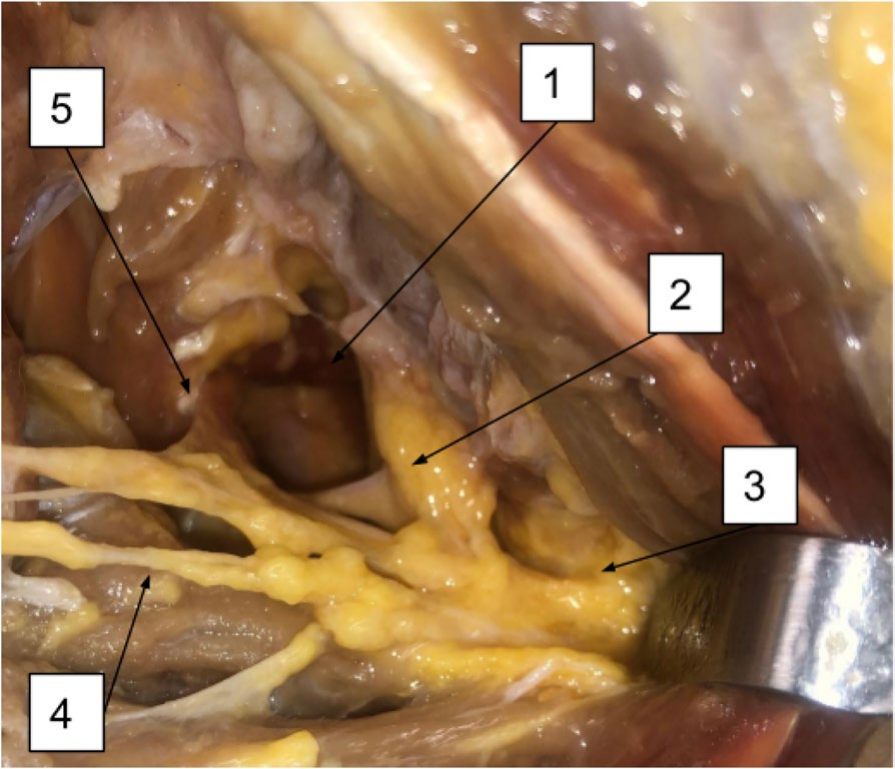
Posterior approach. (1) Quadrangular space; (2) Axillary nerve; (3) Muscular branch to the deltoid muscle; (4) Muscular branch to the teres minor muscle; (5) Articular branch of the axillary nerve

The shoulder joint was then approached dorsally through a transverse incision over the scapular spine. Starting at the lateral border of the acromion to the superomedial angle of the scapula (Fig. 3). A plan was developed between the subcutaneous skin flap and the deep scapular musculature. For the exposure of the supraspinatus muscle, we disinserted the trapezius muscle from the scapular spine. Supra-scapular notch was then palpated to locate the supra-scapular nerve. This was followed by supraspinatus muscle dissection from the supraspinatus fossa. The supra-scapular nerve was exposed till its exit at the spinoglenoid notch (Fig. 4). Access to the infraspinatus and teres minor muscle was facilitated through the detachment of the posterior deltoid fibers from the spine of the scapula. Inferior retraction of the infraspinatus tendon revealed the supra-scapular nerve distal to the spinoglenoid notch, with its muscular and articular branches (Fig. 5). Entering the plane between the deltoid muscle and the teres minor muscle was followed for the identification of the axillary nerve. Axillary nerve exposure was facilitated by following its course from the anterior approach through the quadrangular space just deep to the deltoid (Fig. 6).

## Results

The articular branch of the lateral pectoral nerve, the axillary nerve with its articular branches anteriorly and the subscapularis muscle were all accessible in the cadaveric specimens through the deltopectoral approach. The articular branch of the pectoral nerve course was found just medial and superior to the coracoid process towards the middle of the coraco-clavicular ligament. The axillary nerve course was identified anteriorly till exiting the quadrangular space, its articular nerves to the anterior shoulder capsule were found branching just proximal to the deltoid muscular branches. The subsapular nerve was not dissected, and any possible present articular branches were swept off the capsule by the release of the muscle off the joint capsule.

The articular branches of the supra-scapular nerve and the axillary nerve posteriorly were accessible in all the cadaveric specimens through the proposed posterior approach. The supra-scapular nerve course from the suprascapular notch distally was explored in all the cadavers. An articular branch at the supraspinatus fossa coursing lateral toward the shoulder joint capsule was found arising proximal to the supraspinatus motor branch. Inferior to the spinoglenoid notch, a second articular branch was found coursing laterally in the infra-spinatous fossa toward the posterior shoulder capsule.

The axillary nerve was identified after exiting the quadrangular space posteriorly in all cadavers. Muscular branches were identified piercing the deltoid and teres minor muscles. Articular branches when present were found just proximal to the teres minor muscular branch. One articular branch was found arising from the axillary nerve posteriorly in 4 out of the six cadavers. In one cadaver two articular branches were found. While no articular branches were found posteriorly in one cadaver.

## Discussion

We were able to easily access and interrupt the articular nerve branches to the glenohumeral joint through our proposed anterior and posterior approaches. Primary articular nerves consisting of independent branches from the lateral pectoral nerve, axillary and the supra-scapular nerves were all identified. Accessory articular nerves provided from the sub-scapular nerve were severed by releasing the sub-scapularis muscle off the joint capsule.

In the literature the first description for shoulder joint innervation was postulated in the mid-nineteenth century, in Germany by Nikolaus Rüdinger [12]. This was followed by a large lack of interest for almost a century. Afterwards, new interest by different authors, who had described the contribution of the lateral pectoral nerve, axillary, sub-scapular and the supra-scapular nerve for shoulder innervation [1, 3, 4, 9, 10, 14]. The available studies did not focus on the issue of pain or on surgical approaches for resecting these articular branches.

In the literature we found a single report describing partial shoulder denervation by Dellon [7]. He proposed the resection of a segment of the articular branch of the lateral pectoral nerve through a small incision over the coracoid process, for patients with chronic anterior shoulder pain. The procedure was done after pain relief and increased shoulder range of motion following a local anesthetic block of the branch of the anterior pectoral nerve. However, some authors refrain from using diagnostic blocks when performing joint denervation because the analgesic response after the local block poorly correlated with the postoperative change in pain scores [15]. Furthermore, the local analgesic might spread to smaller terminal nerve branches that are not divided during surgery.

There are no results published for posterior glenohumeral joint denervation. Our proposed technique could help surgeons treat isolated anterior or posterior shoulder pain after failure of other modalites through performing partial denervation for the anterior or the posterior glenohumeral joint. Also, generalized shoulder pain can be treated through total denervation for the whole joint. With the use of intra-operative motor nerve stimulation, differentiation of motor fascicles from articular ones would facilitate the denervation procedure.

Joint denervation has been proven to have no effect on proprioceptive function in animal models [17]. Drawbacks of the procedure include weakness of the trapezius, posterior deltoid and supraspinatus muscles either due the extensive posterior approach or due to interneural plane dissection. In addition to the possible risk of wound problems, painful neuromas and parathesias.

Limitations of the study include its use in cadavers rather than in patients. We believed that the feasibility of the denervation procedure needed to be established prior to performing these surgeries in humans. However, it should be noted that there are no previous studies in the area of performing total glenohumeral denervation, but further studies that include more cases are required to validate this result.

## Conclusion

Total glenohumeral denervation was feasible through our proposed anterior and posterior approaches. Enhanced knowledge of articular nerve branches could provide interventional targets for joint and ligament pain, with low risk of muscle weakness.

## Data Availability

All data generated or analysed during this study are included in this published article.
